# The long noncoding RNA TINCR promotes breast cancer cell proliferation and migration by regulating OAS1

**DOI:** 10.1038/s41420-021-00419-x

**Published:** 2021-03-01

**Authors:** Die Lu, Shihao Di, Shuaishuai Zhuo, Linyan Zhou, Rumeng Bai, Tianshi Ma, Zigui Zou, Chunni Chen, Miaomiao Sun, Jinhai Tang, Zhihong Zhang

**Affiliations:** 1 Department of Pathology, The First Affiliated Hospital of Nanjing Medical University, 300 Guangzhou road, Nanjing, Jiangsu Province 210029 China; 2 Department of Pathology, Changzhou Jintan District People’s Hospital, Jintan Affiliated Hospital of Jiangsu University, 16 Nanmen Road, Jintan, Jiangsu Province 213200 China; 3 Department of Pathology, Zhejiang Provincial People’s Hospital & People’s Hospital of Hangzhou Medical College, Hangzhou, Zhejiang Province 310014 China; 4 Department of Pathology, the First Affiliated Hospital of Soochow University, 899 Pinghai Road, Suzhou, Jiangsu Province 215000 China; 5 Department of General Surgery, The First Affiliated Hospital of Nanjing Medical University, 300 Guangzhou road, Nanjing, Jiangsu Province 210029 China

**Keywords:** Breast cancer, Cell growth, RNAi

## Abstract

Breast cancer is the leading cause of cancer-related death in women around the world. It is urgently needed to identify genes associated with tumorigenesis and prognosis, as well as to elucidate the molecular mechanisms underlying the oncogenic process. Long noncoding RNAs (lncRNAs) are widely involved in the pathological and physiological processes of organisms and play an important role as oncogenes or tumor suppressor genes, affecting the development and progression of tumors. In this study, we focused on terminal differentiation-induced non-coding RNA (TINCR) (GeneID:257000) and explore its role in the pathogenesis of breast cancer. The results showed that TINCR was increased in breast cancer tissue, and high expression level of TINCR was associated with older age, larger tumor size, and advanced TNM stage. High level of TINCR can promote proliferation and metastasis of breast cancer cells, while downregulation of TINCR induces G1-G0 arrest and apoptosis. Mechanismly, TINCR can bind to staufen1 (STAU1) and then guide STAU1 (GeneID:6780) to bind to OAS1 mRNA (NM_016816.4) to mediate its stability. Thus low level of OAS1(GeneID:4938) can lead to cell proliferation and migration. This result elucidates a new mechanism for TINCR in breast cancer development and provides a survival indicator and potential therapeutic target for breast cancer patients.

## Introduction

Breast cancer is the most common cancer diagnosed among women^1^. Although the treatment of breast cancer has made great progress and the mortality rate has decreased year by year, due to the high incidence of tumor specific death, the prognosis of patients is still worthy of attention^2,3^. There are about 1.7 million new cases every year in the world, accounting for 25% of all new cancer cases among women^4^. According to the data from the Centers for Disease Control and Prevention (CDC)^5^, 245,299 new breast cancer cases occurred in the United States in 2016, and 41,487 women died of breast cancer. According to the data of American Cancer Association^6^, in 2019, there will be 268,600 cases of new invasive breast cancer and 48,100 cases of ductal carcinoma in situ, and 41,760 women are expected to die of breast cancer. Although great progress has been made in the treatment of breast cancer, due to the high tumor specific mortality, the prognosis of patients still deserves attention^2,3^. The molecular mechanism of breast cancer is not clear, so it is urgent to further explore new genes related to the development of breast cancer and clarify the molecular mechanism.

Long noncoding RNA (lncRNA) is a kind of RNA with a length of 200–1,000,000 nt, which cannot encode protein. It is widely involved in the physiological and pathological processes of organisms, and plays a role in the negative or positive feedback loop as an oncogene or tumor suppressor gene^7^.These noncoding RNAs can be used as powerful regulators of various cellular processes, including chromatin modification, alternative splicing, genome rearrangement, gene imprinting, cell proliferation, migration, cell apoptosis, and nuclear cytoplasmic transport. Many studies have shown that, lncRNAs may play a complex and extensive role in the occurrence and development of cancer, and it is hopeful to be a biomarker of cancer diagnosis and potential treatment targets^8^.

TINCR, also known as Linc00036 and PLAC2, is a 3.7 kb lncRNA for terminal differentiation induction. It is located on chromosome 19 of the human genome. TINCR mainly exists in the cytoplasm and participates in differentiation induction, and is necessary for mRNA of key differentiation genes, many of which are mutated in human skin diseases, including ALOXE3, ALOX12B, ABCA12, FLG, LOR, ELOVL3, and CASP14^9^. When TINCR is exhausted, these key differentiation genes are lack of induction, which makes TINCR defective epidermis show abnormal terminal differentiation morphology, which is manifested as human genetic skin diseases with abnormal skin barrier function, such as ichthyosis vulgaris and ichthyosis clown^9–11^. In addition, more and more studies have found that TINCR is abnormally expressed in HCC, lung cancer, bladder cancer, squamous cell carcinoma, breast cancer, prostate cancer, and colorectal cancer, and its expression is closely related to proliferation, apoptosis, invasion, and metastasis, suggesting that TINCR can be used as a biomarker and potential target of treatment^7,12–15^.

However, the research on the expression pattern and mechanism of TINCR in breast cancer is very limited. Some studies have shown that TINCR is HER-2-specific lncRNA, which is significantly upregulated in breast cancer, knocking down TINCR can inhibit the proliferation of breast cancer cells, promote cell apoptosis, and inhibit the process of cell cycle in vitro^2^. Liu Yun et al.^13^ reported that TINCR has tumor promoting activity. They found that TINCR was overexpressed in breast cancer tissues and cell lines, and that high levels of TINCR were associated with a relatively poor prognosis. Therefore, we started to study the expression level of TINCR in breast cancer and tried to clarify the mechanism of TINCR involved in the occurrence and development of breast cancer. This study found that TINCR was significantly upregulated in breast cancer tissues, and the upregulated TINCR was closely related to the age of onset, tumor volume, and TNM stage. Further experiments showed that TINCR promoted the proliferation and metastasis of breast cancer cells by regulating its target gene OAS1. In conclusion, TINCR plays a role of oncogene in breast cancer and can be used as a potential biomarker for diagnosis and treatment.

## Results

### High expression of TINCR in human breast cancer tissues and its clinical significance

**Fig. 1:**
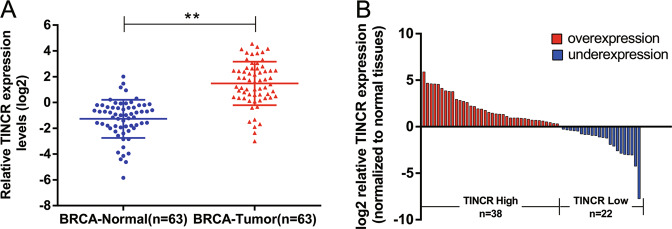
The relative expression of TINCR in breast cancer tissues and its clinical significance. **A** Data from the TCGA database shows the relative expression of TINCR in 63 pairs of breast cancer and adjacent normal tissues. **B** The qRT-PCR experiment detected the relative expression of TINCR in 60 pairs of breast cancer tissues and their adjacent non-cancerous tissues. According to the level of TINCR expression, the tissues were divided into two groups: TINCR high expression group and TNCR low expression group. Error bars indicate mean ± standard errors of the mean. The mean values of two groups were compared via paired, two-tailed Student *t* tests. *P < 0.05, **P < 0.01.

In this study the bioinformatics tool “TANRIC”(http://ibl.mdanderson.org/tanric/_design/basic/ index.html) (data from the Cancer Genome Atlas Database) was used to analyze the expression of TINCR in 63 pairs of human breast cancer tissues. The results showed that TINCR was significantly overexpressed in breast cancer tissues compared to that in adjacent normal tissues (Fig. 1A). Subsequently, we performed qRT-PCR analysis in 60 pairs of breast cancer tissues and found that the expression of TINCR was increased in 38 breast cancer tumor tissues compared to the noncancerous lesions (Fig. 1B). These results suggest that the upregulation of TINCR may be related to the development of breast cancer.

To further explore the role of TINCR in breast cancer, we evaluated the relationship between TINCR expression and clinicopathological characteristics of breast cancer patients. We divided 60 breast cancer patients into TINCR high expression group (*n* = 38) and TINCR low expression group (*n* = 22) (Fig. 1B), according to whether TINCR expression was upregulated or downregulated compared with the corresponding adjacent noncancerous tissue samples. Table 1 shows that TINCR expression level was associated with age (*P* = 0.037), tumor size (*P* = 0.006), and TNM stage (*P* = 0.002). This result indicated that high TINCR expression was probably to be found in patients over 50 years old, and to present larger tumor size and advanced TNM stage. However, there was no significant correlation between TINCR expression and lymphatic metastasis (*P* = 0.118), histological grade (*P* = 0.628), molecular types (*P* = 0.055, *P* = 0.288, *P* = 0.532), and HER2 expression (*P* = 0.122). This result indicates that TINCR expression level is closely related to the pathological stage index of breast cancer patients, which may be a potential target for breast cancer prediction.

**Table 1:** Correlation between TINCR expression and clinicopathological characteristics of breast cancer.

Clinical parameter	TINCR expression		*P*
	High no. cases (*n* = 38)	Chi-squared test *P* value	Chi-squared test *P* value
Age (years)			0.037^*^
≤50	12	13	
>50	26	9	
size			0.006*
≤2 cm	3	9	
>2 cm	35	13	
TNM stage			0.002*
I	3	10	
II + III	35	12	
Lymphatic metastasis			0.118
Yes	20	7	
No	18	15	
Histological grade			0.628
I + II	20	13	
III	18	9	
luminal (A + B) subtype			0.055
Yes	24	19	
No	14	3	
Her2+ subtype			0.288
Yes	9	2	
No	29	20	
Triple negative subtype			0.532
Yes	5	1	
No	33	21	
Her2			0.122
Positive	31	14	
Negative	7	8	

### The influence of TINCR on breast cancer in vitro

#### TINCR promotes proliferation and metastasis of breast cancer cells

**Fig. 2:**
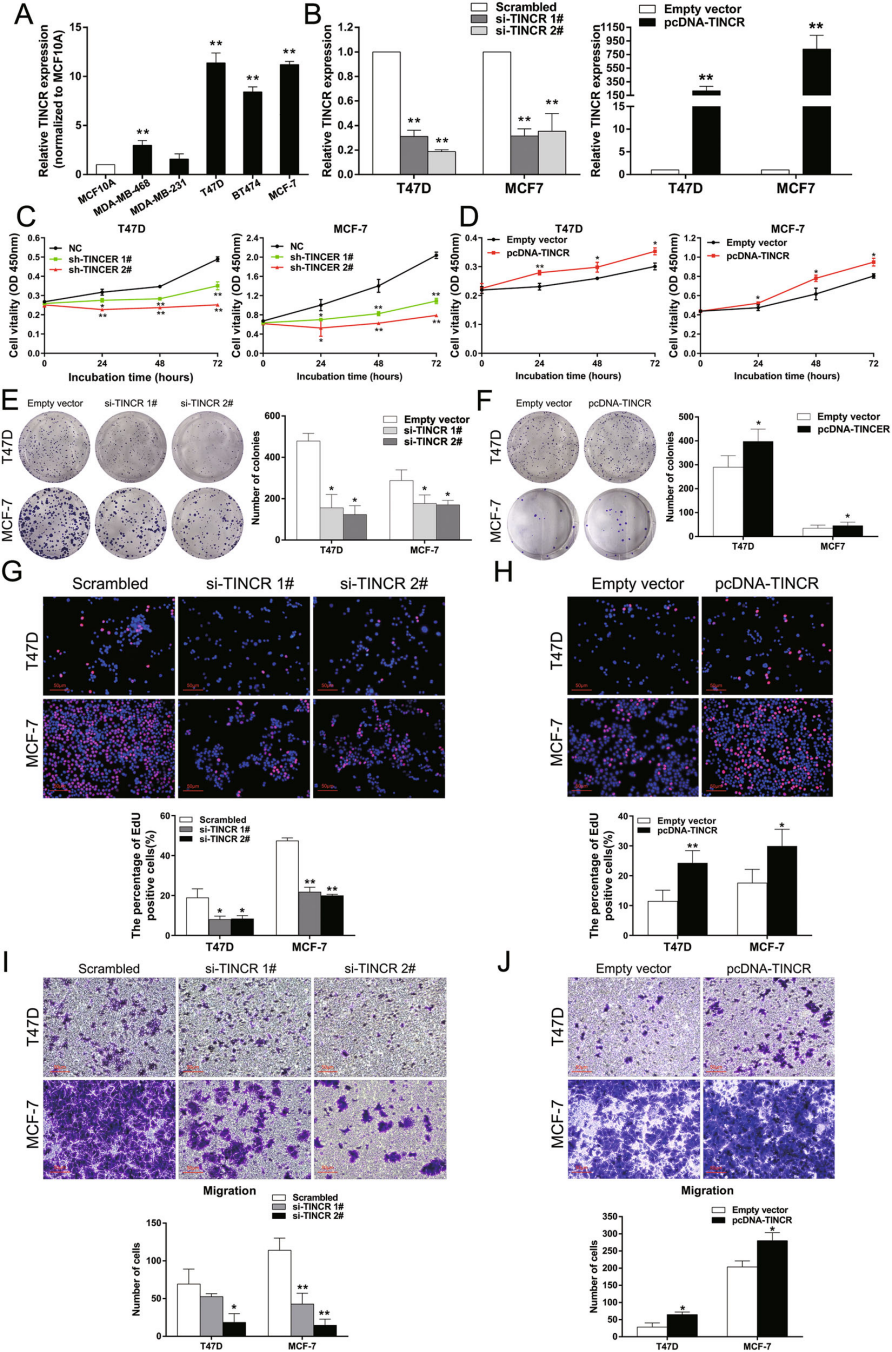
TINCR promotes breast cancer cell proliferation and metastasis in vitro. **A** TINCR expression was examined in normal mammary epithelial cell line (MCF10A) and breast cancer cell lines by qRT-PCR assay. **B** Left: The relative expression of TINCR in T47D and MCF-7 cells transfected with scrambled, si-TINCR 1# and si-TINCR 2#; right: the relative expression of TINCR in T47D and MCF-7 cells transfected with empty vector and pcDNA-TINCR. **C**, **D** CCK-8 assays were performed to determine the viability of T47D and MCF-7 cells treated with si-TINCR or pcDNA-TINCR. **E**, **F** Colony formation assays were used to detect the proliferation of si-TINCR-transfected or pcDNA-TINCR-transfected T47D and MCF-7 cells. Colonies were counted and captured. **G**, **H** Proliferous breast cancer cells were displayed by EdU immunostaining assays. EdU positive cells were counted and captured (400×). **I**, **J** Transwell assays were performed to determine migration in breast cancer cells treated with si-TINCR or pcDNA-TINCR. Values are shown as the mean±s.d in three independent experiments. The mean values of two groups were compared via paired, two-tailed Student *t* tests. *P<0.05, **P<0.01.

To explore the function of TINCR in breast cancer cells, we first performed qRT-PCR analysis to detect its expression level in diverse human breast cancer cell lines. Among them, MCF-7 and T47D belong to luminal type A breast cancer cells (ER + , PR ± , HER2 − ), BT474 belongs to luminal type B (ER + , PR ± , HER2 + ), MDA-MB-468 and MDA-MB-231 belong to triple negative breast cancer cells (ER − , PR − , HER2 − )^16^. These results showed that the expression level of TINCR in breast cancer cells was significantly higher than that in normal breast epithelial cell (MCF10A) (Fig. 2A). Considering the cell culture condition and growth state, T47D and MCF-7 cells were selected for follow-up experiments. Next, two small interfering RNAs (siRNAs) were synthesized to silence TINCR expression. After 48 h of transfection, qRT-PCR analysis showed both si-TINCR 1# and 2# entered the cells effectively and showed efficient interference, which were used in subsequent experiments. Because of the higher interference efficiency of si-TINCR 2#, we designed a short hairpin RNA (shRNA) according to the sequence of si-TINCR 2# for stable transfection. At the same time, we also constructed a pcDNA-TINCR expression vector to upregulate TINCR expression in T47D and MCF-7 cells (Fig. 2B).

As lncRNAs participate in many biological processes, we investigated the influence of TINCR on breast cancer in vitro. Cell counting kit-8 (CCK-8) assay showed that the cell viability of T47D and MCF-7 cells was significantly inhibited after impairing TINCR expression compared to that in control cells (Fig. 2C). In contrast, overexpression of TINCR can significantly improve cell viability (Fig. 2D).In addition, colony formation assays showed that knockdown of TINCR in T47D and MCF-7 cells greatly attenuated clonogenic survival (Fig. 2E), whereas it was significantly increased by upregulation of TINCR expression (Fig. 2F). Similarly, 5-ethynyl-2′-deoxyuridine EdU(red)/DAPI(blue) immunostaining confirmed that proliferation capacity was significantly weakened after inhibition of TINCR expression (Fig. 2G), while overexpression of TINCR had an inverse effect (Fig. 2H).Then, we tested the effect of TINCR on cell metastasis by transwell assay. The results showed that the migration ability of T47D and MCF-7 cells decreased after the inhibition of TINCR expression (Fig. 2I) while overexpression of TINCR enhanced the migration ability (Fig. 2J). The above results confirm that TINCR can promote the proliferation and metastasis in breast cancer cells.

#### Downregulation of TINCR induces G1-G0 arrest and apoptosis of breast cancer cells

**Fig. 3:**
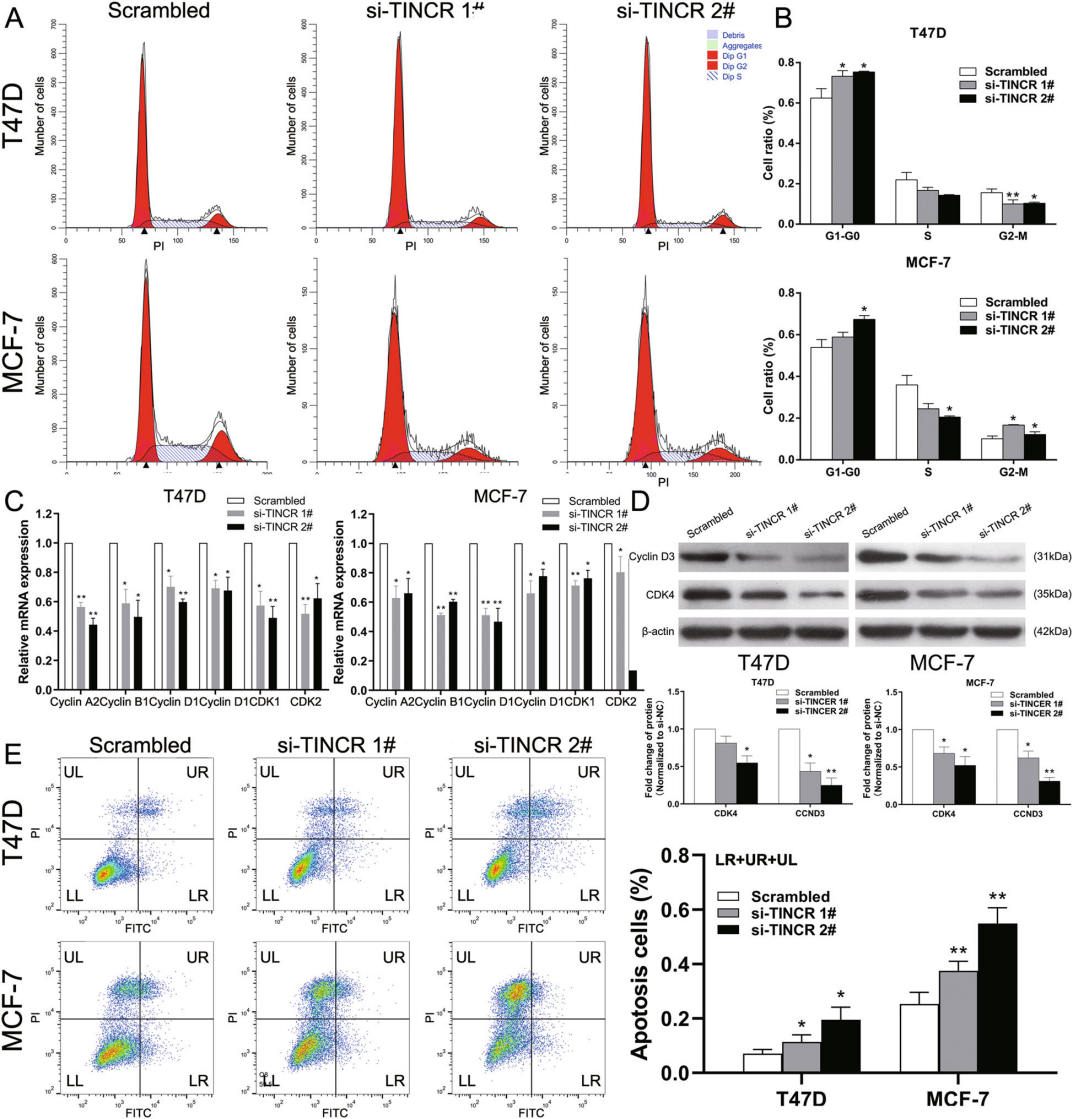
Effects of TINCR on cell cycle and apoptosis of breast cancer cell in vitro. **A**, **B** Cell cycle in breast cancer cells was analyzed by flow cytometry. **C** qRT-PCR analysis of Cyclin A2, Cyclin B1, Cyclin D1, Cyclin D3, CDK1, and CDK2 in breast cancer cells. **D** Western blot analysis of CyclinD3, CDK4 in T47D and MCF-7 cells. β-actin protein was used as an internal control. **E** Apoptotic rates of cells were tested by flow cytometry. LR: early apoptotic cells, UR: terminal apoptotic cells. UL: necrotic cells and terminal apoptotic cells. Values are shown as the mean±s.d in three independent experiments. The mean values of two groups were compared via paired, two-tailed Student *t* tests.*P<0.05, **P<0.01.

Cell growth is generally regulated by cell cycle and apoptosis process. Therefore, flow cytometry was performed to evaluate these factors. The results showed that T47D and MCF-7 cells transfected with si-TINCR 1# and 2# had G1-G0 arrest compared to the control group (Fig. 3A, B). Then, we performed qRT-PCR to detect the mRNA level of cell cycle related genes. The results showed that the expression of Cyclin A2, Cyclin B1, Cyclin D1, Cyclin D3, CDK1, and CDK2 decreased significantly after the knock down of TINCR(Fig. 3C), while western blot proved the decrease in protein level of CDK4 and Cyclin D3 (Fig. 3D),indicating that TINCR is involved in cell cycle regulation, and the decreased levels of Cyclin D1, Cyclin D3, and CDK4 confirmed that TINCR knockdown can cause G1-G0 arrest. Flow cytometry revealed that the proportion of apoptotic cells treated with si-TINCR 1# and 2# was remarkably increased in breast cancer cells (Fig. 3E). These results confirmed that the proliferation inhibition of breast cancer cells transfected with TINCR siRNAs may be achieved by inducing G1-G0 phase arrest and apoptosis.

### TINCR promotes tumorigenesis of breast cancer cells in vivo

**Fig. 4:**
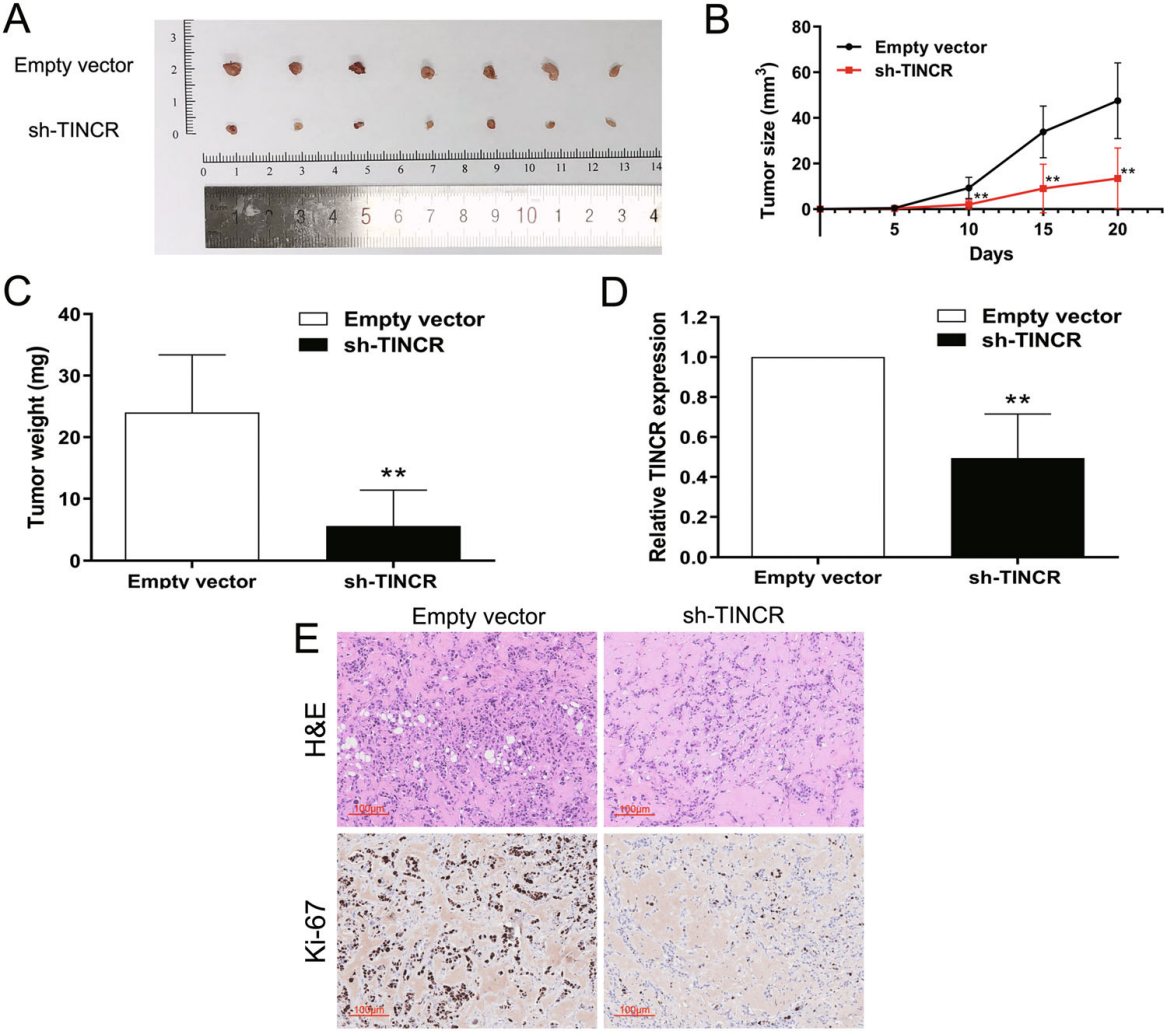
Effects of TINCR on breast cancer tumorigenesis in vivo. **A** Empty vector or sh-TINCR was transfected into MCF-7 cells, which were injected in the BALB/c-nude mice (*n*=7), respectively. Tumors before and after carrying from the nude mice. **B** Tumor volumes were calculated after injection every five days. Points, mean (*n*=7); bars indicate SD. **C** Tumor weights were represented as means of tumor weights±SD. **D** qRT-PCR was used to detect the average expression of TINCR in xenograft tumors (*n*=7). **E** The xenograft tumor sections were under H&E staining and immunohistochemistry staining using antibodies against ki-67 (200×). Values are shown as the mean±s.d in three independent experiments. The mean values of two groups were compared via paired, two-tailed Student *t* tests.*P<0.05, **P<0.01.

In order to further evaluate the tumor-promoting effect of TINCR in vivo, a xenograft tumor model was constructed. MCF-7 cells transfected with empty vector or sh-TINCR were subcutaneously inoculated into the armpits of nude mice. Xenograft tumors appeared at the injection site of all mice. After 20 days of injection, the nude mice were euthanized by spinal cord amputation and the subcutaneous tumor was removed (Fig. 4A). The results showed that mice in the sh-TINCR group grew smaller subcutaneous tumors than those in the control group (Fig. 4B). Tumor growth in the sh-TINCR group was substantially slower than that in the empty vector group and the sh-TINCR group showed lower tumor weights as well (Fig. 4C). qRT-PCR analysis also revealed that tumor tissues in the sh-TINCR group expressed lower TINCR levels than those of the control group (Fig. 4D). Ki-67 levels can reflect proliferation ability. Immunohistochemical analysis showed that Ki-67 staining of subcutaneous tumor tissue in sh-TINCR group displayed lower intensity than that in control group (Fig. 4E).

### Mechanism of TINCR regulating the proliferation and metastasis of breast cancer cells

#### TINCR can bind to STAU1

**Fig. 5:**
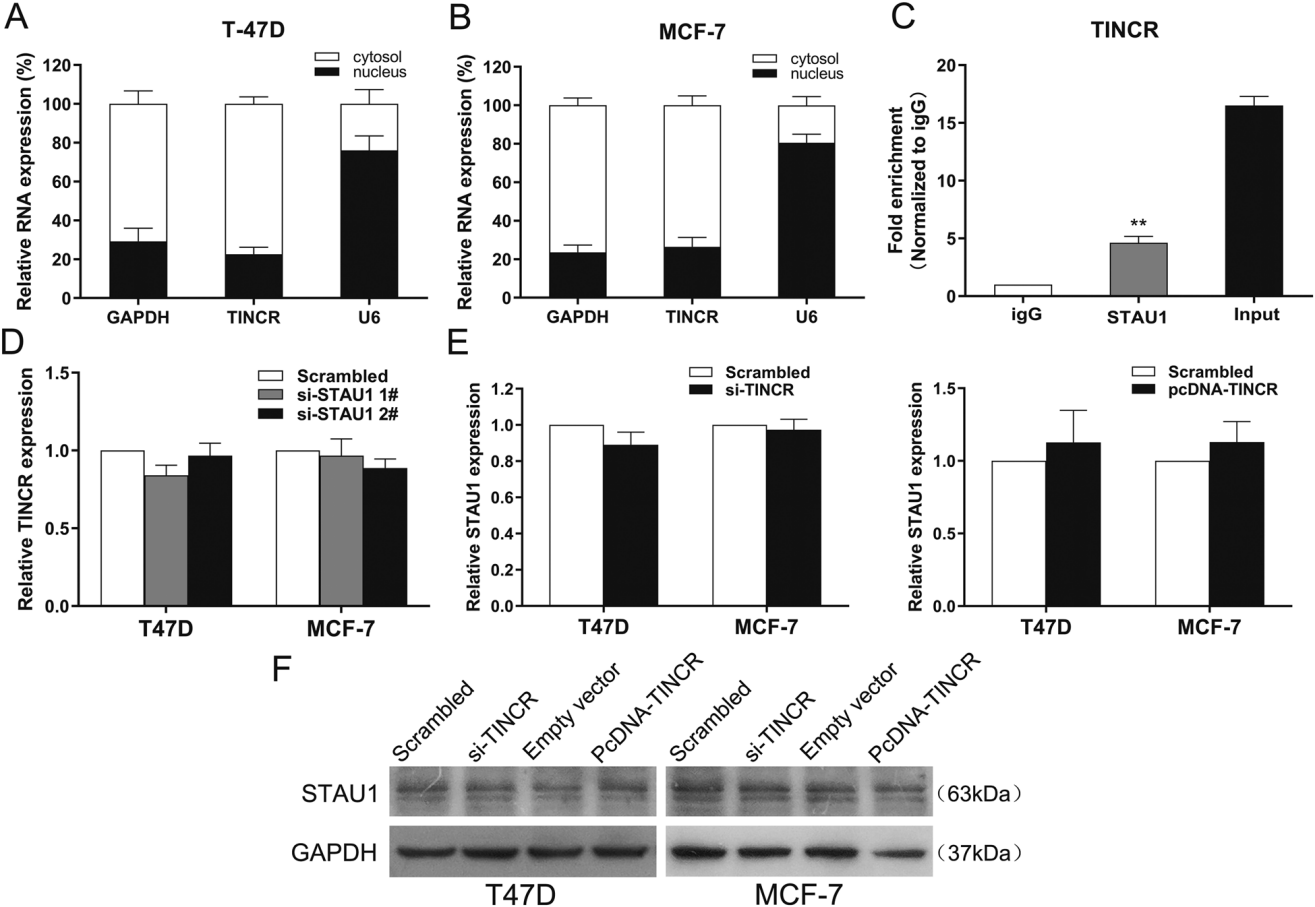
TINCR can bind to cytoplasmic protein STAU1. **A,** **B** qRT-PCR assays were performed to reveal the expression levels of TINCR in the cytoplasm or nucleus of T47D and MCF-7 cells. GAPDH was used as a cytosol marker and U6 was used as a nuclear marker. **C** RIP assay was performed with IgG or STAU1 antibodies in T47D cells, and the coprecipitated RNA was subjected to qRT-PCR for TINCR. **D** qRT-PCR assay examined TINCR expression in breast cancer cells transfected with control (scrambled), si-STAU1 1# and si-STAU1 2#. **E** qRT-PCR assay examined STAU1 expression in breast cancer cells treated with si-TINCR or pcDNA-TINCR. **F** Western blot assay examined STAU1 expression in breast cancer cells treated with si-TINCR or pcDNA-TINCR. Values are shown as the mean ± s.d in three independent experiments. The mean values of two groups were compared via paired, two-tailed Student *t* tests.*P < 0.05, **P < 0.01.

To further explore the mechanism of TINCR in breast cancer, we first determined the distribution of TINCR in breast cancer cells. Previous studies have revealed that^10,12,17,18^ TINCR can directly bind to staufen1 (STAU1) through a 25-nucleotide motif (TINCR box), and then guide STAU1 to bind to specific mRNAs to mediate the stability of differentiated mRNAs. This process is called Staufen-mediated mRNA decay (SMD). STAU1 binds to STAU1-binding site (SBS), which was formed when Alu elements in lncRNAs directly bind to the Alu elements in the 3′-untranslated region (3′UTR) of mRNA via base-pairing rules, in its target mRNA 3′-untranslated region (3′UTR) to induce degradation of target mRNA^19^. Alu elements, once were thought of as “junk” DNA, are now proven to function within RNAs to regulate primate gene expression by diverse mechanisms, for example, triggering SMD^20^. Nuclear and cytoplasmic separation assay showed that TINCR was distributed in the cytoplasm of T47D and MCF-7 cells (Fig. 5A, B), suggesting that TINCR is likely to bind STAU1 protein, which is mainly expressed in cytoplasm, and has a posttranscriptional regulation function. Furthermore, RNA IP revealed significant TINCR enrichment by STAU1 antibody compared with the IgG control (Fig. 5C).We also found that after using siRNA-STAU1 to knock down the STAU1 level in breast cancer cells, there was no significant change in the expression level of TINCR (Fig. 5D), and there was no significant change in the RNA/protein level of STAU1 in TINCR knockdown/overexpression cells (Fig. 5E, F), indicating that TINCR can directly bind with STAU1 without influencing its expression level.

#### Combination of TINCR and STAU1 reduces the stability of OAS1

**Fig. 6:**
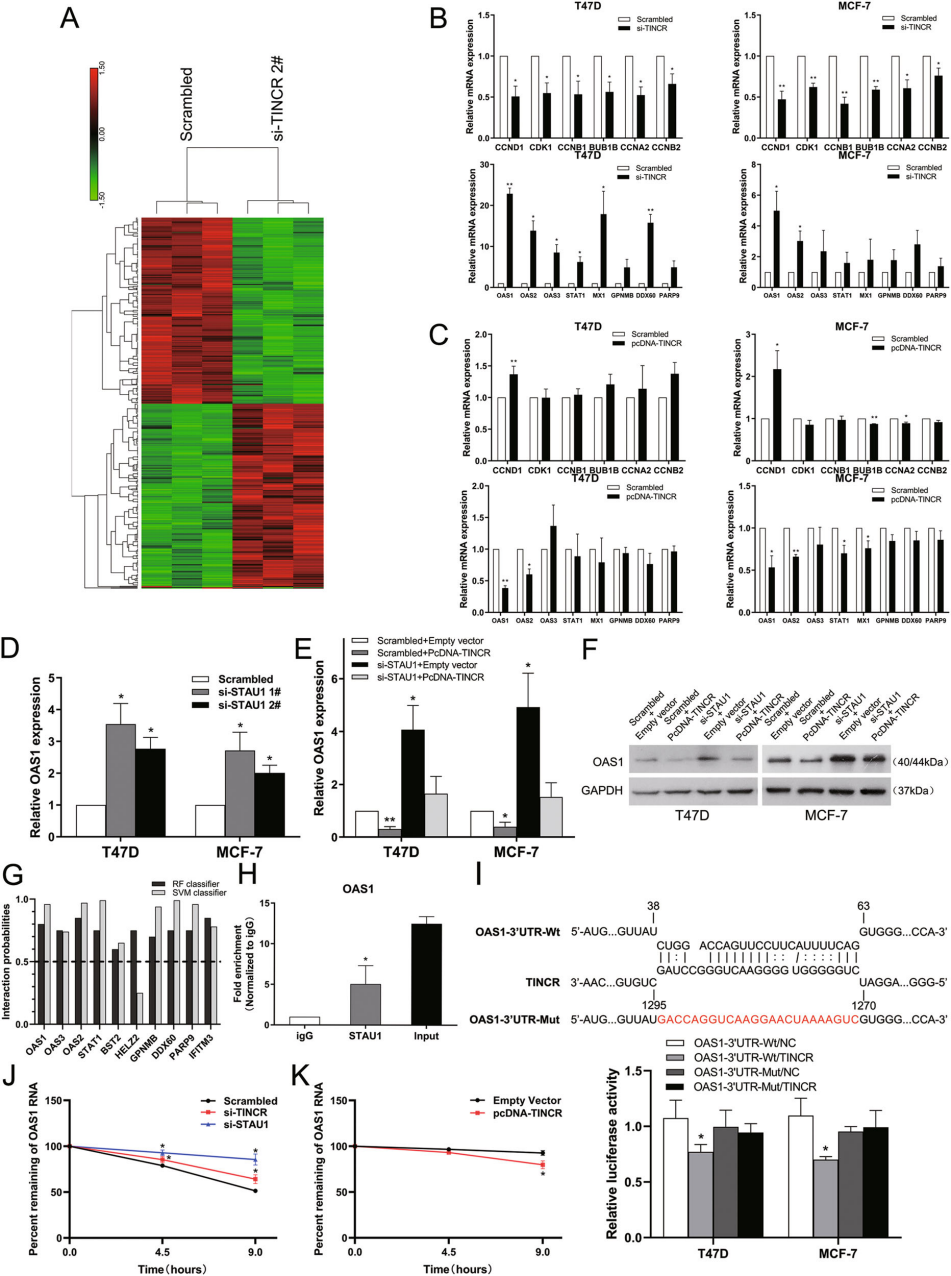
TINCR undermine the stability of OAS1 through binding to STAU1. **A** Mean centered, hierarchical clustering of transcripts altered in breast cancer cells treated with scrambled siRNA or si-TINCR 2#, with three repeats. **B**, **C** qRT-PCR analysis was used to validate the changes of several top regulated mRNAs upon TINCR depletion and overexpression in T47D and MCF-7 cells. **D** OAS1 mRNA expression upon STAU1 depletion, as detected by quantitative reverse transcription (qRT–PCR). **E** OAS1 mRNA expression was detected by qRT–PCR in pcDNA-TINCR and si-STAU1 co-transfected T47D and MCF-7 cells. **F** Protein level of OAS1 was detected by western blot assays in pcDNA-TINCR and si-STAU1 co-transfected T47D and MCF-7 cells. **G** Bioinformatics tool (http://bioinfo.bjmu.edu.cn/lncpro/) was used to evaluate the combination possibility between several top upregulated mRNAs and STAU1. Predictions with probabilities >0.5 were considered “positive”, indicating that the corresponding RNA and protein are likely to interact. OAS1 showed high possibility to interact with STAU1. **H** RIP assay was performed with IgG or STAU1 antibodies in T47D cells, and the coprecipitated RNA was subjected to qRT-PCR for OAS1. **I** The predicted TINCR binding site in OAS1 (OAS1-3′UTR-Wt) and the designed mutant sequence (YY2-30 UTR-Mut) are shown (up). Luciferase reporter gene assays of T47D and MCF-7 cells are shown (down) (OAS1-3′UTR-Wt + TINCR-NC group is seen as control). **J**, **K** TINCR and STAU1 control OAS1 mRNA stability. RNA stability assays were performed in T47D cells using Actinomycin D to disrupt RNA synthesis, and the degradation rates of the OAS1 mRNA were measured over 9 h. Values are shown as the mean ± s.d in three independent experiments. The mean values of two groups were compared via paired, two-tailed Student *t* tests.*P < 0.05; **P < 0.01.

In purpose to determine the target genes regulated by TINCR, we used RNA transcriptome sequencing (RNA-seq) technology to detect the gene expression profiles of breast cancer cells in which TINCR expression was suppressed. Control (Scrambled) and si-TINCR 2# were transfected into T47D cells respectively, then the RNA was sent for examination 48 h after transfection. Sequencing results showed that 280 genes were upregulated (Fold change > 2, *P* value < 0.05) and 282 genes were down regulated (Fold change > 2, *P* value < 0.05; Fig. 6A).

In order to verify the accuracy of the transcriptome sequencing results, we used qRT-PCR to detect the expression levels of some of the most obvious upregulated and downregulated genes in T47D and MCF-7 cells. The results showed that the expression of CCND1, CDK1, CCNB1, BUB1B, CCNA2, and CCNB2 decreased, while the expression of OAS1, OAS2, OAS3, STAT1, MX1, GPNMB, DDX60, and PARP9 increased (Fig. 6B). These results approximately matched the RNA-seq results, suggesting that these differentially expressed genes may be potential downstream targets of TINCR. In addition, our qRT-PCR assays also showed OAS1 (2′-5′-oligoadenylate synthetase 1) mRNA was the most upregulated/downregulated transcript among the transcripts that were commonly regulated upon TINCR downregulation/upregulation, corresponding to our RNA sequencing data (Fig. 6C).

In order to investigate whether OAS1 mRNA is degraded by SMD, we used qRT-PCR to detect the effect of knocking down STAU1 on the expression of OAS1 in breast cancer cells. The results showed that the expression level of OAS1 was significantly increased in T47D and MCF-7 cells knocked down by STAU1 (Fig. 6D). Further experiment revealed that RNA/protein level of OAS1 was regulated both by TINCR and STAU1 (Fig. 6E, F). Then we use bioinformatics method to predict the binding possibility between STAU1 and OAS1. The results show that there is a high binding possibility between STAU1 and OAS1 (Fig. 6G). RIP assay also revealed significant OAS1 enrichment by STAU1 antibody compared with the IgG control (Fig. 6H). Furthermore, bioinformatics analysis was performed by Guangzhou RiboBio Co., Ltd.(Guangzhou, China) to identify the putative SBS between TINCR and OAS1.Then, luciferase assays were used to precisely identify the SBS to clarify the association between the Alu element of TINCR and the 3′-UTR of OAS1 (Fig. 6I), the results showed that relative luciferase activity in the OAS1-3′ UTR-wild-type (WT) + TINCR group was significantly decreased compared with NC group, whereas there was no such difference between the OAS1-3′ UTR-mutant-type (Mut) + TINCR and control groups. Based on the above data, we speculate that OAS1 may be an important downstream gene regulated by TINCR, and TINCR can reduce its stability by combining with STAU1.

In order to further study whether TINCR–STAU1 complex can regulate the stability of OAS1 mRNA, we transfected siRNA-TINCR and siRNA-STAU1 into T47D and MCF-7 cells, then treated these cells with actinomycin D (ACTD). Cells were collected every 4.5 h to extract RNA for qRT-PCR assay. We found that compared with the control group, the mRNA level of OAS1 was significantly increased after TINCR or STAU1 was knocked out, whereas the mRNA level of OAS1 was significantly lower than that of the control group after TINCR was overexpressed, (Fig. 6J, K). In conclusion, these results show that TINCR can reduce the stability of OAS1 by combining with STAU1.

#### Inhibition of carcinogenesis of TINCR by OAS1

**Fig. 7:**
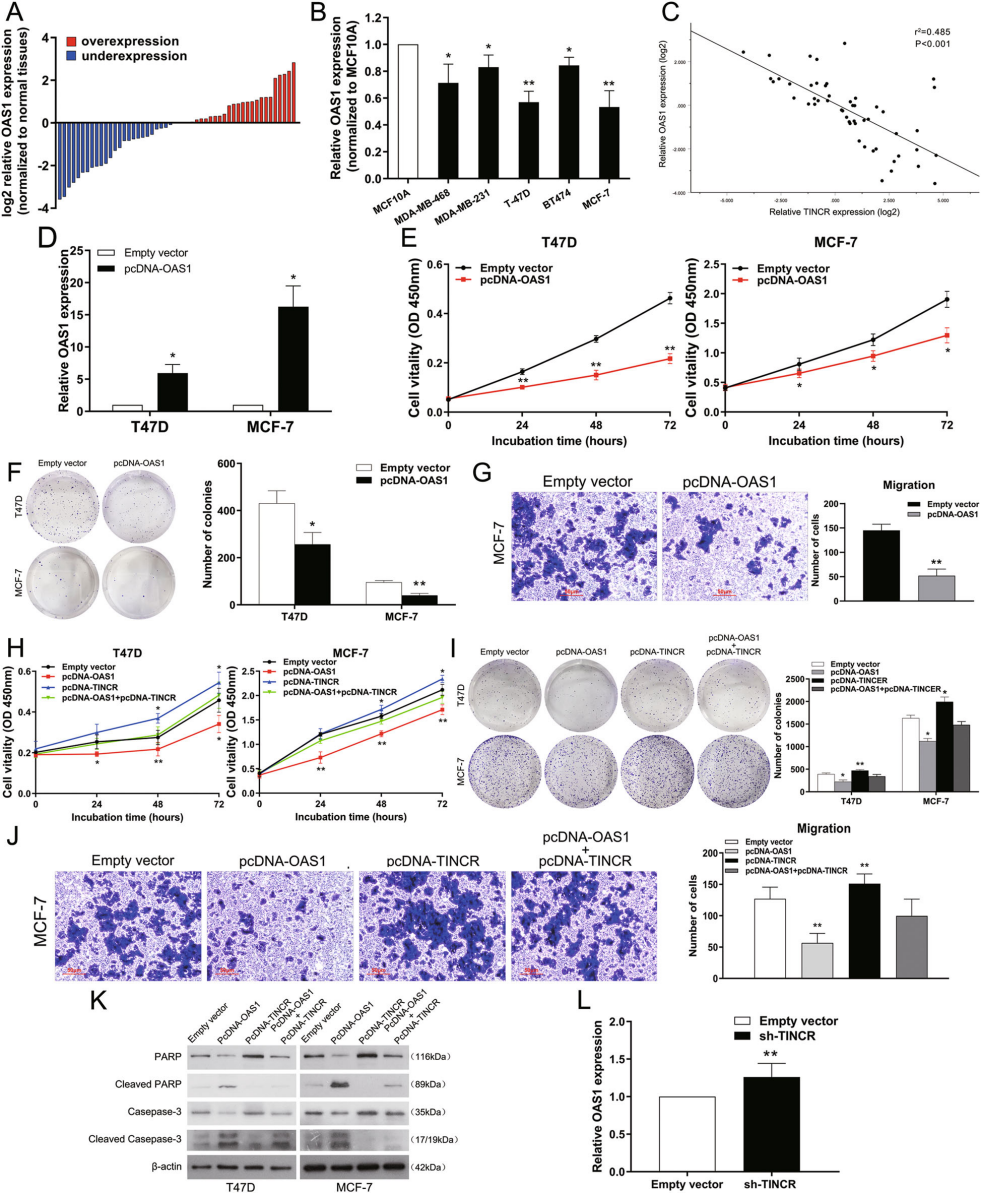
Increased expression of OAS1 inhibits breast cancer cell proliferation and is involved in the oncogene function of TINCR. **A** qRT-PCR assays were conducted in 51 paired human breast cancer tissues and their corresponding adjacent non-tumor tissues to evaluate relative expression of OAS1, and the result was normalized against GAPDH expression. **B** qRT-PCR analysis of OAS1 expression in normal mammary epithelial cell line (MCF10A) and breast cancer cells. **C** Correlation analysis of TINCR expression (log2 value) and OAS1 mRNA level (log2 value) in 51 breast cancer tissues using Pearson’s correlation coefficient. **D** qRT-PCR analysis of OAS1 expression in T47D and MCF-7 cells transfected with control (empty vector), pcDNA-OAS1. **E**, **F** The viability of T47D and MCF-7 cells transfected with pcDNA-OAS1 or control (empty vector) was determined by CCK-8 and colony formation assays. Experiments were performed in triplicate. **G** Migration ability was investigated by transwell assays in MCF-7 cells transfected with pcDNA-OAS1 or control (empty vector). **H**, **I** CCK-8 and colony formation assays showed the cell viability of pcDNA-OAS1 and pcDNA-TINCR co-transfected T47D and MCF-7 cells. Experiments were performed in triplicate. **J** Migration ability was investigated by transwell assays in pcDNA-OAS1 and pcDNA-TINCR co-transfected MCF-7 cells. **K** Western blot analysis of apoptosis-related proteins in T47D and MCF-7 cells co-transfected with pc-DNA TINCR, pcDNA-OAS1, or empty vector. β-actin protein was used as an internal control. **L** qRT-PCR was used to detect the average expression of OAS1 in xenograft tumors (*n*=7). Values are shown as the mean±standard errors of the mean from three independent experiments. The mean values of two groups were compared via paired, two-tailed Student *t* tests. *P<0.05, **P<0.01.

To further explore the role of OAS1 in breast cancer, we first detected the level of OAS1 in 51 pairs of breast cancer tissues and five breast cancer cell lines. The results showed that compared with the adjacent noncancerous tissues and normal breast epithelial cells, the level of OAS1 in breast cancer tissues and cell lines decreased significantly (Fig. 7A, B). Further analysis revealed that TINCR expression was inversely correlated with OAS1 level in breast cancer (Pearson correlation coefficient *r* = −0.697, *r*^2^ = 0.485, *P* < 0.001; Fig. 7C).

Then we synthesized the pcDNA of OAS1 to detect the function of OAS1 (Fig. 7D). CCK-8 and colony formation assays showed that overexpression of OAS1 could weaken cell proliferation (Fig. 7E, F). Transwell assays confirmed that upregulation of OAS1 could inhibit cell migration (Fig. 7G). These results demonstrated upregulation of OAS1 inhibited the proliferation and migration of breast cancer cells, similar to the results of TINCR knockdown.

In addition, we used rescue experiments to explore whether OAS1 is involved in the carcinogenic effect of TINCR in breast cancer. We co-transfected pcDNA-TINCR and pcDNA-OAS1 in T47D and MCF-7 cells. CCK-8 and colony formation experiments showed that overexpression of OAS1 could partially rescue pcDNA-TINCR-induced cell proliferation (Fig. 7H, I). Transwell assays also showed that overexpression of OAS1 could partially rescue cell migration induced by TINCR overexpression (Fig. 7J). Moreover, western blot assays revealed that OAS1 can induce cell apoptosis which can also be rescued by TINCR overexpression. As showed in Fig. 7K the protein levels of cleaved PARP and cleaved caspase-3 were increased in breast cancer cells treated with pcDNA-OAS1, while decreased after we added pcDNA-TINCR. In conclusion, these results suggest that TINCR can promote cell proliferation and migration as well as inhibit cell apoptosis by downregulating the expression of OAS1. Moreover, qRT-PCR assay revealed that knocking down TINCR could elevate the expression of OAS1 in the xenograft tumors (Fig. 7L). These data confirm that TINCR can contribute to the malignant phenotype by inhibiting OAS1.

## Discussion

LncRNA TINCR has been proved to be abnormally expressed in a variety of tumors, which may play an oncogenic or tumor suppressor role and affect the occurrence and development of tumors^21^. Several studies have shown that TINCR is significantly upregulated in breast cancer and plays as an oncogene, and its upregulated expression is believed to be mainly caused by gene amplification^2,13,22^. In this study, we for the first time analyzed the relationship between TINCR and the clinicopathological characteristics of breast cancer patients, as well as verified the binding between TINCR and STAU1 in breast cancer cells. Our study identified a new downstream target of TINCR-OAS1 and found that OAS1 mRNA stability can be mediated through SMD caused by TINCR-STAU1 combination.

TINCR is a key lncRNA for somatic tissue differentiation. Previous studies have showed that TINCR play critical roles in multiple biological processes implicated in the development and progression of cancers. In lung cancer, TINCR can suppress cell proliferation and invasion through regulating miR-544a/FBXW7 axis^23^. While, high level of TINCR can induce cell proliferation by affecting KLF2 mRNA stability in gastric cancer^12^. Our study focused on the function and mechanism of TINCR in breast cancer. We found that TINCR expression was upregulated in breast cancer tissues and cell lines. Furthermore, high expression of TINCR was related to higher malignant degree. Previous studies also found TINCR was linked to poor prognosis^2,13^. These results indicate that TINCR can be used as a diagnostic as well as prognosis marker of breast cancer. In order to explore the oncogene role of TINCR in breast cancer, we carried out the function deletion/expression experiment. In vitro and in vivo experiments showed that overexpression of TINCR can promote the proliferation of breast cancer cells, and this effect may be achieved by regulating cell cycle and apoptosis. At the same time, overexpression of TINCR can enhance the migration ability of cells.

In order to elucidate the mechanism of TINCR, we clarified OAS1 as a potential downstream target of TINCR through RNAseq. OAS1 is characterized by its ability to catalyze the synthesis of 2–5-linked adenosine oligomers from adenosine triphosphate (2–5A). 2–5A binds to potential ribonuclease L (RNase L), which then dimers into its active form^24–26^. Inhibition of RNase L activity in cancer cell lines can significantly inhibit apoptosis, that is, RNase L can be used as a strong inducer of apoptosis^27,28^. In our study, we find out that TINCR expression was inversely correlated with OAS1 level in breast cancer tissues. Moreover, OAS1 was significantly increased/decreased in T47D and MCF-7 cells transfected with si-TINCR/pcDNA-TINCR. In another words, TINCR can regulate the expression of OAS1. We also find out that OAS1 is a tumor suppressor gene in breast cancer by inhibiting cell proliferation and metastasis, which can be reversed by TINCR to a certain extent. CLáudio J Maia et al.^27^ also confirmed the low expression of OAS1 in six breast cancer cases. They determined that the immunoreactivity of OAS1 was inversely correlated with the histological grade of breast cancer, suggesting that OAS1 was related to the invasion of breast cancer.

**Fig. 8:**
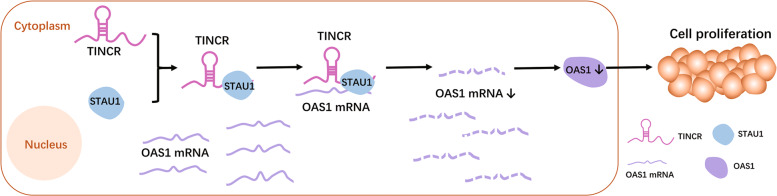
Summary diagram describes that TINCR regulates breast cancer cell proliferation. TINCR-STAU1-OAS1 axis in breast cancer.

We have verified that TINCR can regulate OAS1 in breast cancer, while the mechanism was still not clear. TINCR has been reported to regulate the malignant process of tumor through two mechanisms. First, as the molecular sponge of microRNA (miRNA), it becomes the competing endogenous RNA (ceRNA) of miRNA, modulating target mRNA by competing with miRNA;^13,23,29–31^ the other is to directly combine with proteins in the cytoplasm, such as EpCAM, BRAF, and STAU1, so as to directly affect protein hydrolysis or regulate the stability of downstream target genes through interaction with proteins^12,31–33^. TINCR has been proved to be able to interact with staufen1 (STAU1) located in the cytoplasm through TINCR box and mediate the stability of downstream mRNA through SMD^10,12,18^. With the help of UPF1 and RNA double-strand structure formed by target mRNA and lncRNA, STAU1 can directly bind to target mRNA to induce rapid mRNA degradation^12^. Previous studies and our nuclear cytoplasmic separation experiments as well as RNA IP have confirmed that TINCR mainly exists in the cytoplasm in breast cancer cells, and is involved in posttranscriptional gene regulation by binding with STAU1^21^. RNA IP assay, luciferase assays, and mRNA stability experiments confirmed the combination between STAU1 and TINCR/OAS1, the interaction between the Alu elements in TINCR and OAS1 3′-UTR, as well as verified that OAS1 mRNA stability was affected by the expression level of TINCR and STAU1, which support our speculation that TINCR induced SMD may be the mechanisms of TINCR-OAS1 axis. All the above results indicate that the “TINCR-STAU1-OAS1” axis may play a key role in the progression of breast cancer (Fig. 8). Sp1 and H3K27 have been proved to be involved in inducing TINCR transcription^12,13,22,34^. However, there are few researches on the upstream regulation mechanism of TINCR in breast cancer, it will be one of the focuses of future research. In addition, no study has confirmed that OAS/RNase L pathway is involved in the malignant process of breast cancer. Our research revealed that OAS1 can promote cell apoptosis, while TINCR can partially rescue the promotion. However, whether OAS1 partially inhibits the growth as well as promotes apoptosis of breast cancer cells through OAS/RNase L pathway remains to be supplemented by subsequent experiments.

To sum up, this study identified a new pathway involved in breast cancer development. Our data highlight the significance of this TINCR-STAU1-OAS1 axis in regulating the proliferation and metastasis of breast cancer cells. Our findings enrich the molecular mechanism of TINCR in regulating breast cancer progression. TINCR can be used as a diagnostic marker and treatment target of breast cancer, as well as provide experimental basis for clinical diagnosis and treatment of breast cancer.

## Conclusion

Our study found high expression of lncRNA TINCR in breast cancer and its expression level is related to age, tumor size, and TNM stage. TINCR exerts the role of oncogenes by regulating the cell cycle and gradually promoting the proliferation of retinal cells. OAS1 plays a tumor suppressing role in breast cancer. TINCR binds to the cytoplasmic protein STAU1 and induces the degradation of its downstream target gene OAS1 mRNA through SMD. TINCR can be used as a diagnostic marker and therapeutic target for breast cancer.

## Material and Methods

### Tissue samples and clinical data collection

Our study was in accordance with the Declaration of Helsinki and approved by the Research Ethics Committee of Nanjing Medical University, China. Written informed consent was obtained from all patients. Sixty paired breast cancer and corresponding nontumorous breast tissues were collected from 60 patients at Nanjing Maternity and Child Health Care Hospital (Women’s Hospital of Nanjing Medical University). Before surgery, no patients had received local or systemic treatment. Table 1 is the summary of clinicopathological characteristics of the breast cancer patients. All the collected samples were immediately stored in liquid nitrogen until required.

### Cell culture

Two human breast cancer cell lines (MDA-MB-468, BT474) and the normal human mammary epithelial cell line (MCF10A) were purchased from Shanghai Zhong Qiao Xin Zhou Biotechnology Co.,Ltd. (Shanghai, China). Two breast cancer cell lines (T47D, MCF-7) were purchased from Bena Culture Collection (Kunshan, China). Breast cancer cell line MDA-MB-231 was purchased from Shanghai Genechem Co.,Ltd. (Shanghai, China). T47D, MCF-7, and MDA-MB-231 cells were maintained in Dulbecco’s Modified Eagle Medium (DMEM; GIBCOBRL); BT474 cells were cultured in RPMI-1640 medium (GIBCOBRL); MDA-MB-468 cells were grown in L-15 medium (GIBCOBRL); MCF10A cells were grown in Mammary Epithelial Cell Medium (MEpiCM; ScienCell). DMEM, RPMI-1640 and L-15 were supplemented with 10% fetal bovine serum (FBS; AusGeneX) and antibiotics (100 U/ml penicillin and 100 mg/ml streptomycin) (Invitrogen, Carlsbad, CA, USA). MEpiCM was supplemented with 5% fetal bovine serum (FBS; ScienCell), 2% Mammary Epithelial Cell Growth Supplement (MEpiCGS, ScienCell), and 1% penicillin/streptomycin solution (P/S, ScienCell). All cell lines were cultured in humidified air at 37 °C with 5% CO2 according to the manufacturer’s instructions.

### RNA isolation and qRT-PCR analyzes

TRIZOL reagent (Invitrogen) was used to extract total RNA from tissues or cultured cells. 1 μg RNA was reversely transcribed into cDNA according to the manufacturer’s instructions with the PrimeScript RT Reagent Kit (Takara, Dalian, China). SYBR Premix Ex Taq (Takara) was used for all real-time PCR analyzes. Table S1 listed the specific primers used in this study.

### Transfection

T47D and MCF-7 cells were seeded in 6-well plate before transfection. Small interference RNAs (siRNAs) was transfected using Lipofectamine 2000 (Invitrogen, USA) with the manufacturer’s instructions. Plasmid vector was transfected using FuGENE HD Transfection Reagent (Promega, USA). Two TINCR siRNAs (si-TINCR 1#, 2#) was purchased from Invitrogen. RIBOBIO synthesized STAU1 siRNA and scrambled negative control siRNA (si-NC). Table S2 listed the nucleotide sequences of siRNAs for TINCR and STAU1. Realgene (Nanjing, China) synthesized the overexpression plasmid containing TINCR or OAS1 coding sequences (pcDNA-TINCR) and subcloned it into the pcDNA3.1(+) vector (Invitrogen). After 24 h of transfection, cells were harvested for further functional assays. After 48 h of transfection, cells were subjected to RNA/protein extraction for qRT-PCR or western blot.

### Cell proliferation assays

Cell Counting Kit-8(CCK-8; bimake) was used to detect cell viability. With a density of 3000 cells/well, transfected T47D and MCF-7 cells were seeded onto 96-well plates. Every 24 h, 20 μl CCK-8 was added to each well and then cultured for 2 h to measure cell viability. A certain number of transfected cells were seeded in 6-well plates and cultured for 14 days for colony formation assay. Then methanol was added in each well fix the cells and 0.1% crystal violet (Sigma-Aldrich) was used for staining. In each well, visible colonies were counted.

### Cell Migration Assay

Cell migration ability was evaluated using the cell migration assay with 8 μm pore size polycarbonate membranes (Corning Incorporated). Transfected cells were seeded in 24-well transwell chambers with an 8 mm pore size polycarbonate membrane (Corning Incorporated). Transfected cells was added into the upper chamber with the density of 3 × 10^4^ cells/ml and cultured with serum-free media, while medium containing 10% FBS was added into the lower chamber. After 24 h or 48 h, cells on the lower membrane surface were fixed, stained then imaged using microscope

### Ethynyldeoxyuridine (EdU) analysis

With the density of 2 × 10^4^ cells per well, transfected cells were seeded into 24-well plates. Then, Cell-Light EDU Apollo567 In Vitro Kit (Ribobio, Guangzhou, China) was used for detecting cell proliferation with the manufacturer’s protocol. Under fluorescent microscopy, the percentage of EdU-positive cells was observed and imaged, then calculated from five random fields in three wells.

### Flow cytometric analysis

Forty-eight hours after transfection, transfected T47D and MCF-7 cells were harvested for Flow cytometric analysis using FITC Annexin V Apoptosis Detection Kit (BD Biosciences) for cell apoptosis analysis following the protocol. Then flow cytometry (FACScan^®^; BD Biosciences) with FlowJo_V10 (BD Biosciences) was used for analysis. Cells were classified into different stages and for each experiment the ratio of dead, early apoptotic, and apoptotic cells was compared with the control. Cell Cycle Assay Kit(including RNase A)(FMS-CCC01, FcMACS, Nanjing, China) was used for cell cycle analysis strictly according to the manufacturer’s protocol. ModFit (Verity Software House) was used to analyze the percentage of cells in G0/G1, S, and G2/M phase for further comparation.

### Western blot assay and antibodies

Cells were lysed by RIPA buffer to obtain total protein lysates and then separated by 10% SDS-polyacrylamide gel electrophoresis (SDS-PAGE). After transferred to 0.22 μm NC membranes (Sigma), protein lysates were incubated with specific antibodies. The ECL chromogenic substrate was used to quantify by densitometry (Quantity One software; Bio-Rad). The following primary antibodies were used: Cyclin D3 (Proteintech, no. 26755-1-AP), CDK4 (MXB, no. EP180), OAS1 (D1W3A) Rabbit mAb (Cell Signaling Technology, no. 14498), Cleaved Casepase-3 (Asp175) Antibody (Cell Signaling Technology, no. 9661, PARP (46D11) Rabbit mAb (Cell Signaling Technology, no. 9532), Casepase-3 Antibody (Cell Signaling Technology, no. 9662), Cleaved PARP (Asp214) (D64E10) XP^®^ Rabbit mAb (Cell Signaling Technology, no. 5625), β-Actin Mouse Monoclonal Antibody (Beyotime, no. AF5001), GAPDH Mouse Monoclonal Antibody (Beyotime, no AF5009), Anti-rabbit IgG, HRP-linked Antibody (Cell Signaling Technology, no. 7074S), Anti-mouse IgG, HRP-linked Antibody (Cell Signaling Technology, no. 7076S). The β-actin antibody and GAPDH antibody were used as control.

### Tumor formation assay in a nude mouse model

The study was performed strictly in accordance with the recommendations in the Guide for the Care and Use of Laboratory Animals of the National Institutes of Health. The protocol was approved by the Animal Ethical and Welfare Committee of Nanjing Medical University. Seven 4-weeks-old female BALB/c-nude mice were housed in a specific pathogen-free (SPF) condition and cared with protocols approved by the Shanghai Medical Experimental Animal Care Commission. After transfected with sh-TINCR and empty vector, MCF-7 cells were harvested from 10 cm cell culture dishes, then re-suspended at a density of 1 × 10^8^ cells/ml. Tumor formation were induced by subcutaneously injection of 100 μl single-cell suspension into a single side of each mouse’s armpit. Every 5 days, tumor growth and tumor volumes were examined. Mice were euthanized, and the subcutaneous tumor was taken out at 20 days post-injection for further examination.

### RNA immunoprecipitation (RIP) assay

RIP was performed using the EZ-Magna RIP kit (Millipore, Billerica, MA) following the manufacturer’s protocol. T47D cells at 80–90% confluency were scraped off and then lysed in complete RIP lysis buffer. A total of 100 μl of whole cell extract was incubated with RIP buffer containing magnetic beads conjugated with antibodies against STAU1 or control IgG (Millipore) for 6 h at 4 °C. The beads were then washed with washing buffer, the complexes were incubated with 0.1% SDS/0.5 mg/ml Proteinase K (30 min at 55 °C) to remove proteins. The RNA concentration was measured by NanoDrop spectrophotometer (Thermo Scientific), and the RNA quality was assessed using a bioanalyzer (Agilent, Santa Clara, CA). Finally, immunoprecipitated RNA was purified and analyzed by qRT-PCR.

### Subcellular fractionation location

The separation of nuclear and cytosolic fractions was performed using the PARIS Kit (Life Technologies, Carlsbad, CA) according to the manufacturer’s protocol.

### Immunohistochemical (IHC) analysis

The primary tumors were immune-stained for Ki-67 as previously depicted. Mouse anti-Ki-67 monoclonal antibody was used to evaluate the Ki-67 labeling index, which was calculated as the proportion of positive tumor nuclei divided by the total number tumor cells examined.

### Dual-luciferase reporter assay

Bioinformatics analysis was performed by Guangzhou RiboBio Co., Ltd.(Guangzhou, China) to identify the potential TINCR binding sites of OAS1 3′-UTR. The 3′-UTR fragment of OAS1 with the theoretical TINCR binding site was constructed to form the pmiR-RB-Report^TM^ vector OAS1-3′-UTR-wild-type (IRF6-3’-UTR-Wt) (RiboBio). To mutate the putative binding site of TINCR in the 3′-UTR-containing vector, the sequence of the putative binding site was replaced as indicated and formed the OAS1-3′-UTR-mutated-type (OAS1-3’-UTR-Mut). Wild-type vector or mutated-type vector was transfected into T47D and MCF-7 cells using Lipofectamine 3000 (Invitrogen, USA). Relative luciferase activities were measured 48 h after transfection and renilla luciferase activity was normalized by firefly luciferase activity.

### Statistical analysis

All statistical analyzes were performed using SPSS 17.0 software (IBM, SPSS, USA). The results from at least three independent tests are shown as the mean value ± standard deviation (SD). The significance of differences between groups was estimated by a paired, two-tailed Student’s *t* test or *χ*2 test as appropriate. *P* values less than 0.05 were recognized as significant.

## Supplementary information

table S1

table S2
