# Supplementary material for: The long noncoding RNA TINCR promotes breast cancer cell proliferation and migration by regulating OAS1
Source: Cell Death Discov. 2021 Mar 1;7:41. doi: 10.1038/s41420-021-00419-x (PMC7921111; doi:10.1038/s41420-021-00419-x)
Supplement: Supplementary file 1 — table S1 [file 41420_2021_419_MOESM1_ESM.docx]

Table S1

| Gene | Forward primer | Reverse primer |
| --- | --- | --- |
| TINCR  OAS1  STAU1  OAS2  OAS3  STAT1  GPNMB  DDX60  PARP9  GAPDH | TGTGGCCCAAACTCAGGGATACAT  TCGGACGGTCTTGGAATTAG  CATGCTGGAGATCCTTGGTT  CGAGATCCAGAAGTCCCTTG  GAGCAAACCTCCCTCATGC  CAATGCTTGCTTGGATCAGC  CTCTCACGAGCACCCTGATT  TGTACGTTGCACCCACAAAG  ATGTCCTGTGCCTCCAACTC  GAAGGTGAAGGTCGGAGTC | AGATGACAGTGGCTGGAGTTGTCA  CACCCAAGTTTCCTGTAGGG  GGCATCCTGAACTCATCCTC  GCCTAGAGGTTGCACAGAGC  CAGCGCAAGATGATGTCAAT  TGTCTTTCCACCACAAACGA  TGCACGGTTGAGAAAGACAC  AAAGCAGGCAGGCACTGTA  TTGCAGAACTGGTATGGGACT  GAAGATGGTGATGGGATTTC |
