# Supplementary material for: The long noncoding RNA TINCR promotes breast cancer cell proliferation and migration by regulating OAS1
Source: Cell Death Discov. 2021 Mar 1;7:41. doi: 10.1038/s41420-021-00419-x (PMC7921111; doi:10.1038/s41420-021-00419-x)
Supplement: Supplementary file 2 — table S2 [file 41420_2021_419_MOESM2_ESM.docx]

Table S2

| Scrambled | ACGUGACACGUUCGGAGAATT |
| --- | --- |
|  | UUCUCCGAACGUGUCACGUTT |
| si-TINCR 1# | GACCUGGGUACUGGCUGAAGGAAUA |
|  | UAUUCCUUCAGCCAGUACCCAGGUC |
| si-TINCR 2# | GGAAAGCACUGUGCCACCUUGGAAA |
|  | UUUCCAAGGUGGCACAGUGCUUUCC |
| si-STAU1 1# | GAGAACAUGCUGGAGAUCCUUGGUU |
|  | AACCAAGGAUCUCCAGCAUGUUCUC |
| si-STAU1 2# | CAAGGCCACGGUAACUGCCAUGAUA |
|  | UAUCAUGGCAGUUACCGUGGCCUUG |
| sh-TINCR | CACCGGAAAGCACTGTGCCACCTTGGAAATTCAGAGATTTCCAAGGTGGCACAGTGCTTTCC |
|  | AAAATTTCCAAGGTGGCACAGTGCTTTCCTCTCTGAAGGAAAGCACTGTGCCACCTTGGAAAC |
